# Optimising (re-)irradiation for locally recurrent head and neck cancer: impact of dose-escalation, salvage surgery, PEG tube and biomarkers on oncological outcomes—a single centre analysis

**DOI:** 10.1186/s13014-024-02570-y

**Published:** 2025-01-02

**Authors:** Julia Katharina Schleifenbaum, Janis Morgenthaler, Shachi Jenny Sharma, Jens Peter Klußmann, Philipp Linde, Simone Wegen, Johannes Rosenbrock, Christian Baues, Emmanouil Fokas, Richard Khor, Sweet Ping Ng, Simone Marnitz, Maike Trommer

**Affiliations:** 1 Department I of Internal Medicine, Faculty of Medicine and University Hospital Cologne, University of Cologne, Cologne, Germany; 2 Centre for Integrated Oncology Aachen Bonn Cologne Duesseldorf, Cologne, Germany; 3 Department of Radiation Oncology, Cyberknife and Radiotherapy, Faculty of Medicine and University Hospital Cologne, Cologne, Germany; 4 Department of Otorhinolaryngology, Head and Neck Surgery, Faculty of Medicine, University of Cologne, Cologne, Germany; 5 Department of Radiation Oncology, University Hospitals of the Ruhr University of Bochum, Ruhr University Bochum, Bochum, Germany; 6 Department of Radiation Oncology, Olivia Newton-John Cancer Wellness and Research Centre, Austin Health, Melbourne, VIC Australia; 7 Privatpraxis für Radioonkologie im Vosspalais, Voßstr. 33, 10117 Berlin, Germany

**Keywords:** Recurrent head and neck cancer, Reirradiation, Salvage therapy, PEG tube, Inflammation, Biomarkers

## Abstract

**Introduction:**

Locoregional recurrence (LR) is common in locally advanced head and neck cancer (HNSCC), posing challenges for treatment. We analysed outcome parameters and toxicities for patients being treated with radiotherapy (RT) for LR-HNSCC and investigated patient and disease related prognostic factors in this prognostically unfavourable group.

**Methods:**

This analysis includes 101 LR-HNSCC patients treated with RT, radio-chemotherapy (RCT) or radio-immunotherapy (RIT) between 2010 and 2018 at a high-volume tertiary centre. Patient characteristics, tumour and treatment details were retrospectively collected. Overall survival (OS), progression-free survival (PFS) and toxicities according to Common Terminology Criteria for Adverse Events (CTCAE) v5.0 were assessed.

**Results:**

62% of patients were radiotherapy-naïve (initial RT group) while 38% were re-irradiated at site of LR (re-RT group). Median OS for initial RT was 24 months, for re-RT 12 months (*p < *0.01). In the RCT subgroup, patients with initial RT had significantly longer OS with 35 months compared to re-RT 12 months (*p < *0.05). Patients with UICC grade IV tumours and percutaneous endoscopic gastrostomy (PEG) tube had significantly shorter OS in multivariate analysis: initial RT 13 vs. re-RT 32 months and initial RT 12 vs. re-RT 32 months respectively. Salvage surgery before RT at recurrence was a positive prognostic factor for OS (initial RT 35 vs. re-RT 12 months). Other significant factors for longer OS in univariate analysis included low inflammatory status (Glasgow Prognostic Score 0) and radiation doses ≥ 50 Gy. We detected 37 (15%) ≥ CTCAE Grade 3 events for initial RT and 19 (15%) for re-RT patients.

**Conclusion:**

In this analysis, we identified key prognostic factors including PEG tube and inflammation status that could guide treatment decision. Our findings suggest salvage surgery as preferred treatment option with postoperative RT at LR. Adverse events due to re-RT were acceptable. A radiation dose of ≥ 50 Gy should be administered to achieve better outcomes.

**Supplementary Information:**

The online version contains supplementary material available at 10.1186/s13014-024-02570-y.

## Introduction

Cancer of the head and neck remains a challenging tumour entity, with a high incidence of locoregional recurrence (LR) or second primary cancer even after multimodality treatment. Locoregional failure occurs in up to 52% of patients with locally advanced head and neck squamous cell carcinoma (HNSCC) [1–3]. For LR cases, treatment options include salvage surgery or re-irradiation (re-RT), with or without chemotherapy, as recommended by national guidelines [4–6]. However, salvage surgery is often limited by the recurrence’s proximity to pre-irradiated or previously operated areas [7, 8]. Chemotherapy has traditionally been the standard of care for patients ineligible for surgery. Despite using multi-drug regimens like EXTREME or TP-EXTREME, response rates remain modest at around 35%, with long-term disease control being rare [9, 10]. The advent of immune checkpoint inhibitors (ICI) targeting the programmed cell death (ligand) 1 (PD-1) and PD-L1 pathways has improved overall survival (OS) significantly in patients with LR and/or metastatic HNSCC, particularly those with PD-L1-positive tumours [11]. However, even with these advances, up to 83% of patients experience disease progression within the first year of treatment [11–13]. Radiotherapy (RT) is an efficient and potentially curative alternative treatment option for LR-HNSCC but also challenging due to the increased risk of severe treatment-related toxicities and potential tumour radio-resistance especially at re-RT [14–16]. While studies suggest that combining re-RT with salvage surgery can improve disease-free survival, this approach results in significant acute and late toxicities [17]. For patients who are not surgical candidates, re-RT, with or without chemotherapy, could offer a potential pathway to long-term survival in carefully selected cases [18]. Despite these insights, the evidence for the role of initial RT and re-RT in the management of LR-HNSCC remains limited. This scarcity of data complicates the development of robust treatment algorithms, especially for patients with high-risk or recurrent disease. To address this gap, our retrospective study analysed outcomes and toxicities in patients treated with RT for LR-HNSCC at our facility. By investigating patient- and disease-related prognostic factors, we aim to identify variables associated with improved outcomes, ultimately contributing to more effective treatment strategies for this challenging patient group.

## Methods

### Patients and parameters

**Fig. 1:**
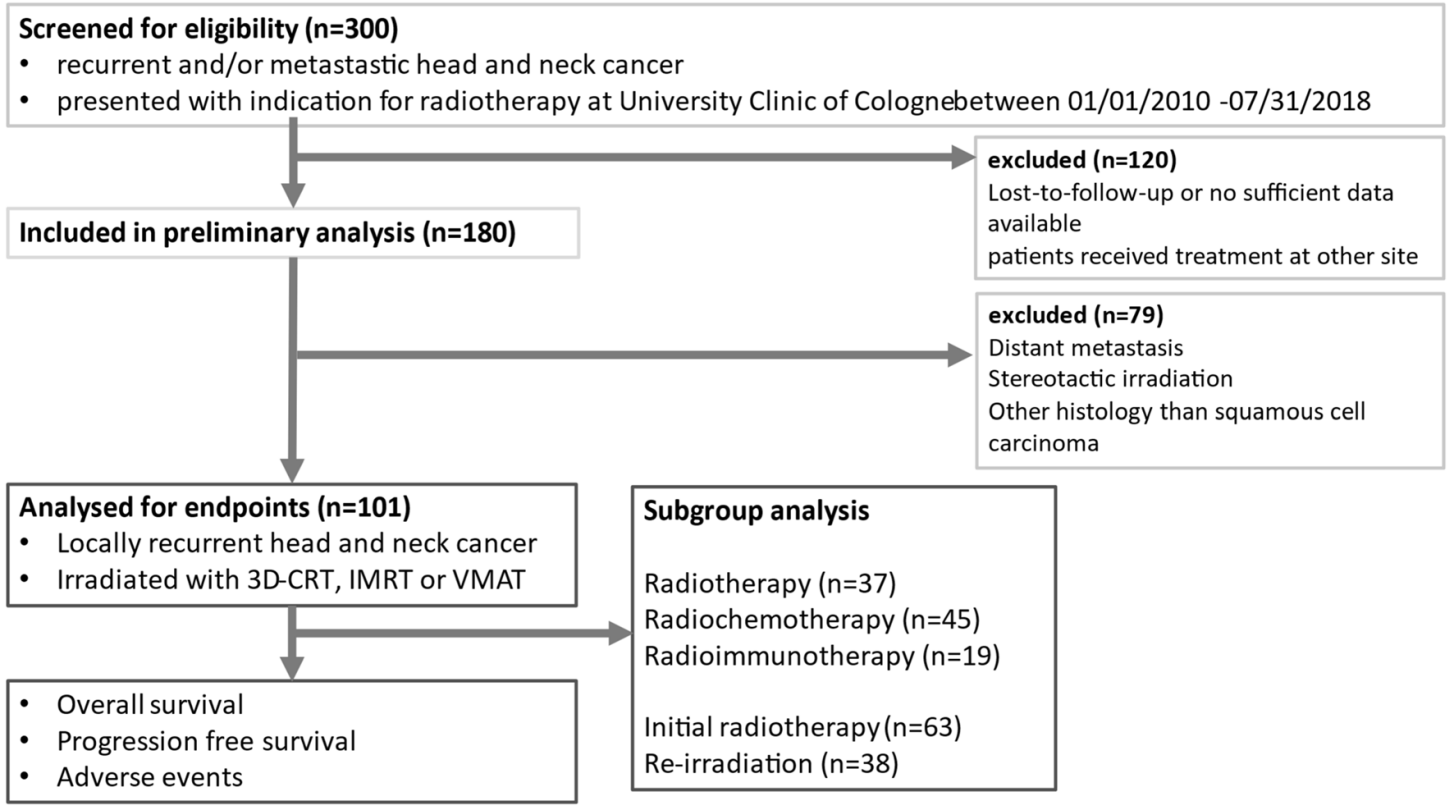
Inclusion and exclusion criteria for patients with locoregional recurrence of head and neck squamous cell carcinoma (LR-HNSCC) treated at the department of radiotherapy at University Clinic of Cologne, Germany. 3D-CRT: 3D-conformal radiotherapy IMRT: intensity-modified radiotherapy VMAT: volumetric intensity modified arc therapy

The cohort was defined by screening records of patients treated at the University Hospital Cologne between January 2010 and July 2018 for LR-HNSCC. We included patients with recurrent SCCs of the head and neck who received RT, radio-chemotherapy (RCT), or radio-immunotherapy (RIT) with cetuximab. We divided these patients into two groups: initial RT was defined as RT applied to patients without any prior irradiation (initial RT group) and re-RT was defined as RT applied to patients with prior irradiation at the site of local recurrence (re-RT group). Patients were excluded if they had histology other than SCC, distant metastases, other malignancies and/or received systemic chemotherapy only. Data were retrospectively collected. For inclusion/exclusion criteria and study conduct, see Fig. 1. Patient characteristics were collected including sex, age, tumour characteristics, Eastern European Oncology Cooperation performance score (ECOG-PS), nutritional status, smoking and alcohol consumption status, and relevant pre-treatment and treatment data. From blood specimens, we obtained C-reactive protein (CRP) and albumin for calculating the high-sensitive modified Glasgow Prognostic Score (hsmGPS), which is an independent, systemic inflammation-based prognostic value based on C-reactive protein (CRP) and albumin (Score 0 = CRP ≤ 3 mg/l, any albumin; Score 1 = CRP > 3 mg/l, albumin ≥ 35 g/l; Score 2 = CRP > 3 mg/l, albumin < 35 g/l) [19–21]. The study was approved by the ethics committee of the University Hospital of Cologne (20-1108).

### Interventions

Patients presented at our centre for RT at diagnosis of LR-HNSCC either with inoperable tumours for definitive RT or after salvage surgery for adjuvant RT. Salvage surgery was performed before whenever possible. Initial RT and re-RT were planned using 3D-CRT or IMRT/VMAT aimed at target dose of 66 Gy and higher with normo- or hypofractioned approaches. For delineation of primary tumour clinical target volume (CTV) and nodal station, the consensus guidelines from Gregoire et al. were used [22, 23]. OAR constraints for primary radiotherapy according to the Quantitative Analysis of Normal Tissue Effects in the Clinic (QUANTEC) initiative and its updates were applied [24–30]. Dose equivalent of 2 Gy was calculated using the linear quadratic model with an α/β- constant of 10 Gy for tumour tissue, mucosa, and acute complication [31]. For patients who received re-RT, a cumulative DVH was created using EQD2 for normal tissues using both the previous RT dose and the current. A tissue-dependent recovery or dose discount (i.e., the amount of the previously given dose that is assumed to be recovered and can be subtracted for further cumulative dose calculations) was not used. Target volumes were prioritised and the doses to the OAR were guided by the patient’s life expectancy, risk acceptance, and the general treatment goal. In cases where the OAR doses were exceeded, irreversible toxicities after previous RT were considered and discussed with the patient. This approach is in consensus with the recent European Society for Radiotherapy and Oncology (ESTRO) and European Organisation for Research and Treatment of Cancer (EORTC) recommendations on re-RT [32]. No patients were re-irradiated if the period from completion of the first RT series to the recurrence was less than 6 months. Chemotherapy was given with cisplatin mono or platinum-based combinations with 5-FU or paclitaxel as treating physician’s choice. For patients unsuitable for platinum-based treatment, recommendations as per Ahn et al. were applied [33]. Immunotherapy was applied via epidermal growth factor receptor (EGFR) inhibitor cetuximab (400 mg/m^2^ loading dose and 250 mg/m^2^ weekly). Patients were treated with primary or adjuvant RT depending on operability. All patients received dietary counselling before start of therapy. If there was a concern that the patient might not keep up sufficient oral intake during therapy or if the tumour was of obstructive nature, a PEG tube was recommended if it had not already been placed due to medical need.

### Endpoints

Primary endpoint of this study included OS; secondary endpoints included progression-free survival (PFS) and adverse events. OS was defined as the time from start of initial RT or re-RT until death, PFS as the time from start of initial RT or re-RT to further recurrence or progression event. Adverse events or toxicity were graded using the Common Terminology Criteria for Adverse Events version 5.0 (CTCAE) [34].

### Statistical analysis

All analyses were performed using SPSS version 27.0 (IBM Corp. Released 2020. IBM SPSS Statistics for Macintosh, version 27.0. Armonk, NY: IBM Corp), R-Studio R-4.3.1 or Microsoft Excel, version 16.54. P-values of < 0.05 were considered statistically significant. Categorical variables are reported with absolute and relative frequencies, continuously normally distributed variables with mean and standard deviation, continuously nonnormally distributed variables with median and interquartile range (IQR). For comparison of categorical variables, Pearson-Chi-2 test for contingency analysis with two-sided significance or Fisher's exact test with two-sided significance were used. Post-hoc analysis was performed with Bonferroni tests. For noncategorical variables Mann–Whitney U test for two-level variables and Kruskal–Wallis test for multilevel variables were used. For survival analysis (OS and PFS), Kaplan–Meier method was applicated. When proportional hazards were given, log-rank procedure (Mantel-Cox) was added. For metric variables we used univariate Cox regression, for comparison of covariates we performed multivariate cox regression model. A maximum of one variable per 10 events was included in multivariate cox regression model.

## Results

### Patient and treatment characteristics

101 patients with LR-HNSCC patients were eligible for analysis of OS, PFS and adverse events. For the conduct of the study, see Fig. 1. For the detailed descriptive analysis, see Supplementary Data Table 1. We performed group comparison for initial RT und re-RT, demonstrated in Table 1. Patient groups were mainly well balanced. Patients in the initial RT group received a significantly higher EQD2 radiation dose than in the re-RT group (*p < *0.001). PTV in the re-RT group was significantly smaller than in the initial RT group (*p < *0.001), lymph drainage region was significantly less extensive in the re-RT group (*p* =  0.001). Salvage surgery was performed significantly more often in the initial RT group (*p < *0.001) and curative treatment intent was also significantly more common in this group (*p* =  0.002).

**Table 1:** Group comparison of initial RT and re-RT for patients with locoregional recurrence of head and neck squamous cell carcinoma (LR-HNSCC)

		iRT (*n* = 63)	re-RT (*n* = 38)	*p*-value
		n (%) / med (IQR)	n (%) / med (IQR)	
Patient characteristics				
Sex	Male	46 (73.0)	28 (73.7)	0.941^a^
	Female	17 (27.0)	10 (26.3)	
Age		64.5 (13.25)	60 (11.25)	0.338^c^
ECOG-PS	0	28 (62.2)	12 (60.0)	0.864^b^
	1	10 (22.2)	6 (30.0)	
	2	5 (11.1)	1 (5.0)	
	3	2 (4.4)	1 (5.0)	
BMI	< 18.5	7 (14.0)	3 (9.4)	0.099^b^
	18.5–24.9	19 (38.0)	20 (62.5)	
	> 25.0	24 (48.0)	9 (28.1)	
Smoking	Yes	33 (63.5)	25 (78.1)	0.158^a^
	No	9 (36.5)	7 (21.9)	
Alcohol consumption	Substantial (C2 abuse)	10 (19.6)	5 (16.7)	0.944^a^
	Occasionally	16 (31.4)	10 (33.3)	
	None	25 (49.0)	15 (50.0)	
Tumour characteristics				
Tumour entity	Oral cavity	32 (50.8)	13 (34.2)	0.084^b^
	Nasopharyngeal	0 (0.0)	1 (2.6)	
	Oropharyngeal	12 (19.0)	11 (28.9)	
	Hypopharyngeal	2 (3.2)	5 (13.2)	
	Salivary glands	1 (1.6)	0 (0.0)	
	Laryngeal	12 (19.0)	5 (13.2)	
	Nasal cavity and paranasal sinus	2 (3.2)	0 (0.0)	
	CUP	1 (1.6)	0 (0.0)	
	Multilevel	1 (1.6)	3 (7.9)	
UICC stage	I	9 (15.5)	0 (0.0)	0.071^b^
	II	4 (6.9)	4 (14.8)	
	III	8 (13.8))	2 (7.4)	
	IV	37 (63.8)	21 (77.8)	
Risk group	Locally limited	13 (22.4)	4 (14.8)	0.415^a^
	Locally advanced	45 (77.6)	23 (85.2)	
Radiation characteristics				
Therapy group	RT	27 (42.9)	10 (26.3)	0.078^a^
	RCT	28 (44.4)	17 (44.7)	
	RIT	8 (12.7)	11 (28.9)	
Technique	IMRT	15 (23.8)	12 (32.4)	0.617^a^
	VMAT	19 (30.2)	9 (24.3)	
	3D-CRT	29 (46.0)	16 (43.2)	
EQD2_10Gy_				
Dose per fraction	Gy	62.84 (10.62)	50.4 (11.42)	< 0.001*^c^
	Gy	1.8 (0.2)	1.8 (0.2)	
PTV	ccm	598.55 (793.07)	222.8 (272.8)	< 0.001*^c^
Inclusion of lymph drainage region	Yes	44 (69.8)	14 (36.8)	0.001*
	No	19 (30.2)	24 (63.2)	
Additional therapies				
Chemotherapy	Cisplatin/Carboplatin mono	16 (59.3)	5 (31.3)	0.077^b^
	Cisplatin/5-FU	3 (11.1)	0 (0.0)	
	Carboplatin/Paclitaxel	6 (22.2)	9 (56.3)	
	Others	2 (7.4)	2 (12.5)	
Salvage surgery	Yes	33 (53.2)	33 (86.8)	< 0.001*^a^
	No	29 (46.8)	5 (13.2)	
Curative intent	Yes	51 (82.3)	19 (50.0)	0.002*^a^
	No	11 (17.7)	19 (50.0)	

^a^Chi-square test, ^b^Fisher's exact test; ^c^Mann–Whitney U test for independent samples; *significant. Significant if *p < *0.05. Reported n are valid n. iRT, initial RT; Med, median; IQR, interquartile range; ECOG-PS, Eastern Cooperative Oncology Group Performance Score; BMI, body mass index; RT, radiotherapy; RCT, radio-chemotherapy; RIT, radioimmunotherapy; IMRT, intensity-modulated RT; VMAT, Volumetric Intensity Modulated Arc Therapy; 3D-CRT, three-dimensional conformational RT; EQD2, equivalent dose of 2 Gy, PTV, planned target volume.

### Survival outcomes (OS, PFS) for different therapy groups

**Fig. 2:**
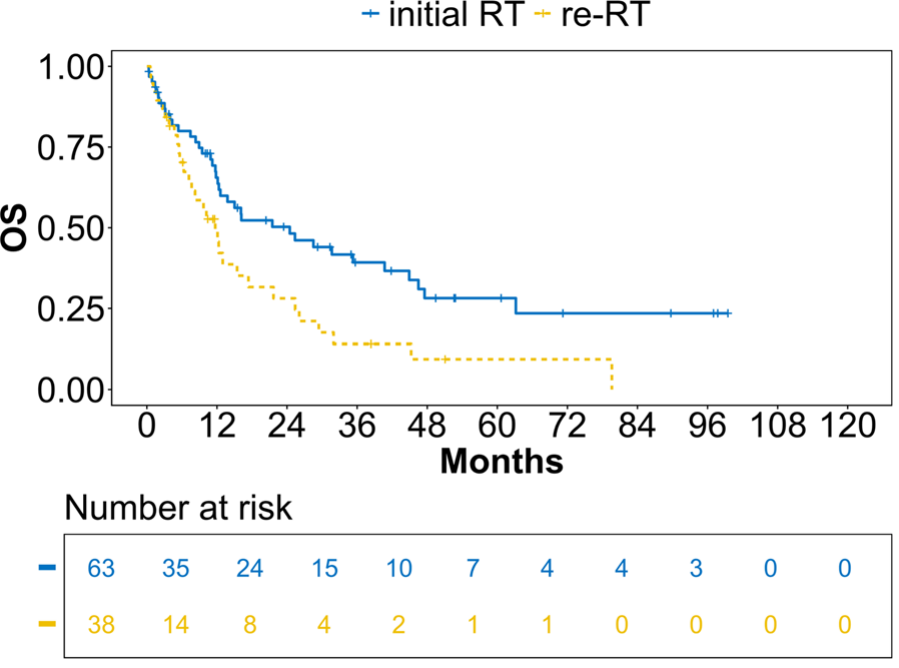
OS estimates for initial RT/re-RT for patients with locoregional recurrence of head and neck squamous cell carcinoma (LR-HNSCC). Valid *n* =  101, analysis according to Kaplan–Meier, *p*  =  0.013. OS = overall survival

**Fig. 3:**
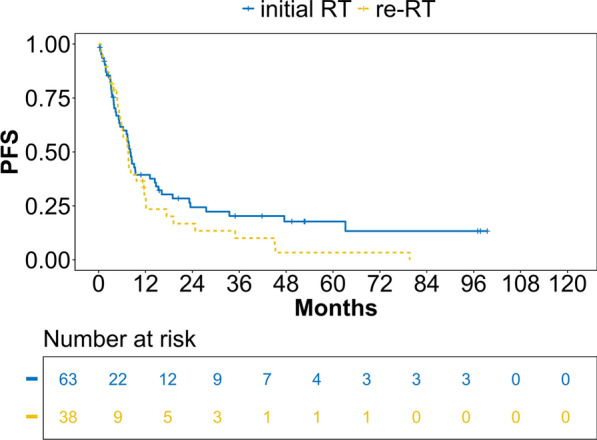
PFS estimates for initial RT/re-RT for patients with locoregional recurrence of head and neck squamous cell carcinoma (LR-HNSCC). Valid *n* =  101, analysis according to Kaplan–Meier. PFS = progression-free survival

**Fig. 4:**
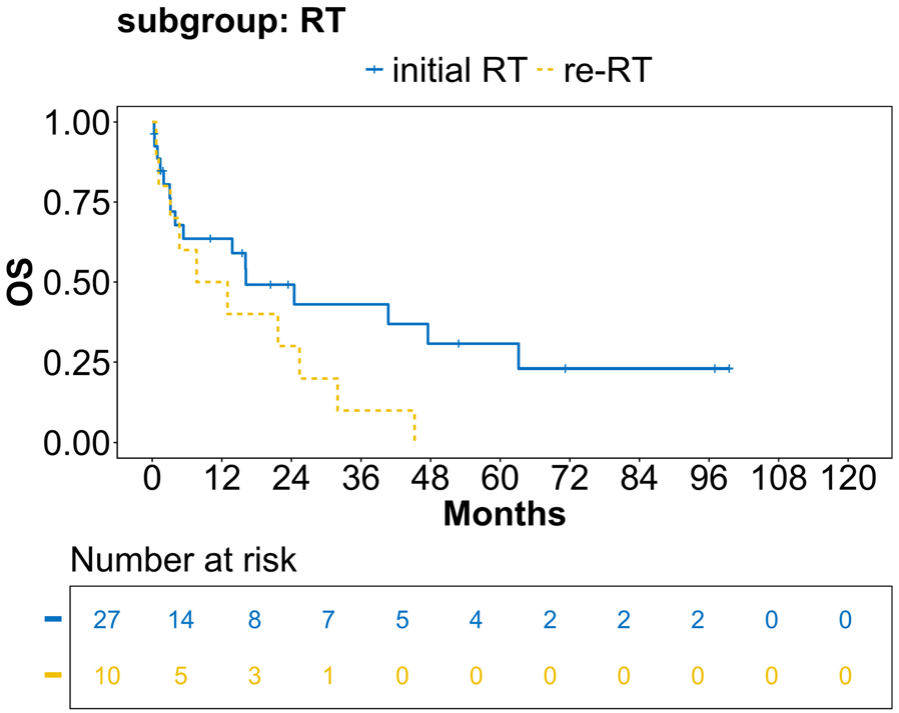
OS estimates for initial RT/re-RT for patients with locoregional recurrence of head and neck squamous cell carcinoma (LR-HNSCC) receiving RT alone. Valid *n* =  37, analysis according to Kaplan–Meier. OS = overall survival

**Fig. 5:**
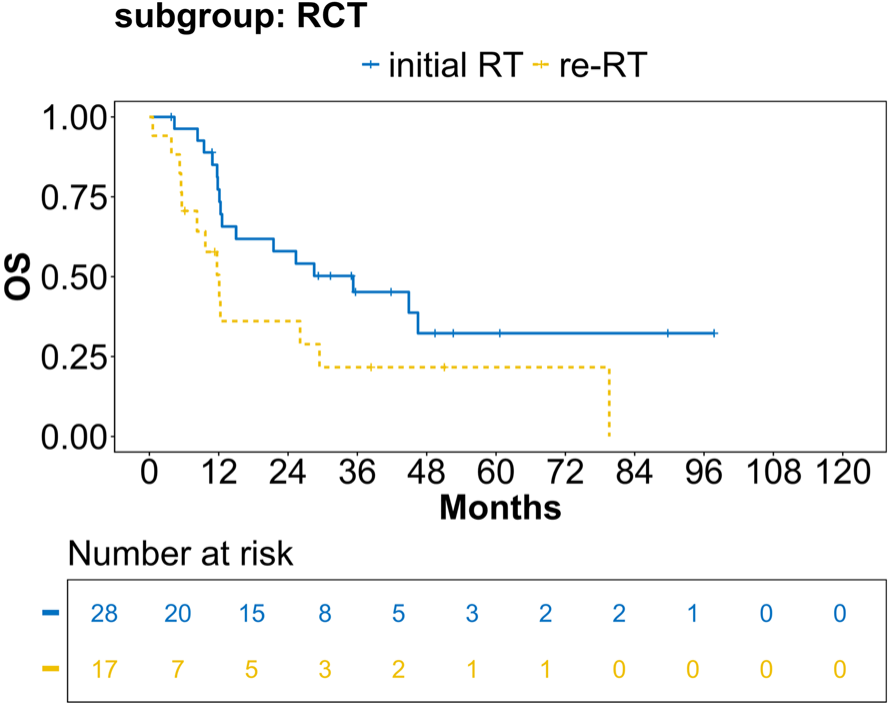
OS estimates for initial RT/re-RT for patients with locoregional recurrence of head and neck squamous cell carcinoma (LR-HNSCC) receiving combined RCT. Valid *n* =  45, analysis according to Kaplan–Meier, *p*  =  0.045. OS = overall survival

Median follow up for all patients was 11 months (IQR 26), median time from first diagnosis of HNSCC to recurrence was 19.5 months (IQR 38.25). OS for LR-HNSCC was 15 months (CI 12.08; 26.05), median PFS was 8 months (CI 6.30; 9.68). Median OS of the initial RT group was 24 months (CI 12.57; 46.43), re-RT 12 months (CI 7.64; 21.66) (*p* =  0.013), see  Fig. 2. Median PFS of the initial RT group was 8 months (CI 6.27; 14.67), re-RT 8 months (CI 5.80; 12.08), see Fig. 3. Regarding the combination of RT with systemic therapy, in the RT only subgroup, median OS was 16 months in the initial RT group (CI 5.38; not reached (NR)), re-RT 10 months (CI 3.12; NR), see Fig. 4. In the RCT subgroup, initial RT patients lived significantly longer than re-RT patients with a median OS of 35 months (CI 12.57; NR) compared to 12 months (CI 8.24; NR) (*p* =  0.045), see  Fig. 5. For RIT, there was not enough data applicable for interpretation. Regarding PFS, there were no significant differences in the RT and RCT subgroups (data not shown).

### Prognostic factors for survival

In the univariate analysis, UICC stage (*p*  =  0.003), PEG tube (*p*  =  0.001), salvage surgery  (*p*  =  0.003), hsmGPS score  (*p*  =  0.035) and EQD2 radiation dose < / ≥ 50 Gy (*p*  =  0.012) correlated significantly with OS. See  Table 2 for detailed data and supplementary data Fig. 1A–E for corresponding Kaplan–Meier curves.

**Table 2:** Survival time analysis of prognostic parameters on OS for all patients with locoregional recurrence of head and neck squamous cell carcinoma (LR-HNSCC)

OS		N	deaths	Censored	median (CI)	*p*-value
UICC stage	Grade I-III	27	14	13	32 (26.05; NR)	0.003*
	Grade IV	58	42	16	13 (10.17; 17.36)	
PEG tube	Yes	54	40	14	12 (8.93; 12.31)	0.001*
	No	45	26	19	32 (24.45; NR)	
Salvage surgery	Yes	34	21	13	35 (16.08; NR)	0.003*
	No	66	47	19	12 (8.24; 17.36)	
hsmGPS	hsmGPS 0	18	12	6	25 (12.27; NR)	0.035*
	hsmGPS 1	36	26	10	12 (9.68; 25.33)	
	hsmGPS 2	4	3	1	2 (1.12; NR)	
EQD2_10Gy_	< 50 Gy	23	17	6	5 (2.66; 26.05)	0.012*
	≥ 50 Gy	76	49	27	21 (12.31; 40.66)	

We performed a Kaplan–Meier analysis per category. We supplemented parameters for which proportional hazards were given with the log-rank procedure. Numbers refer to valid values. Significant results (*p < *0.05) are marked with *PEG: percutaneous endoscopic gastrostomy, hsmGPS: high-sensitive modified Glasgow prognostic score, EQD2, Dose equivalent to 2 Gy; OS, overall survival; CI, confidence interval; NR, not reached

In univariate cox regression model, the following factors were significantly associated with higher risk of death: lower currently applied radiation dose for LR-HNSCC EQD2_10Gy_ < 50 Gy (HR 2.16, CI 1.23; 3.77, *p*  =  0.006), previous radiotherapy (HR 1.87, CI 1.15; 3.04, *p*  =  0.010), hsmGPS score 2 (HR 6.69, CI 1.78; 25.11, *p*  =  0.005), and requiring a PEG tube (HR 2.30, CI 1.38; 3.81, *p*  =  0.001). The following factors were significantly associated with a lower risk of death: higher applied radiation dose at respective irradiation (HR 0.95, CI 0.93; 0.98, *p < *0.001) and conducting salvage surgery (HR 0.47, CI 0.28; 0.79, *p*  =  0.004). We performed a multivariate model (*n* =  81; events 53; degrees of freedom 5) including UICC stage, PEG tube, salvage surgery, previous radiation dose and radiation dose at LR (Table 3).

**Table 3:** Univariate and multivariate Cox proportional hazard model

OS				UV			MV		
		N	death	HR	CI	p-value	HR	CI	p-value
Age		101	68	1.01	0.99; 1.03	0.400			
UICC	grade I-III	27	14	1			1		
	grade IV	58	42	2.40	1.30; 4.43	0.004*	2.11	1.11; 4.02	0.023*
PEG tube	Yes	54	40	2.30	1.38; 3.81	0.001*	2.92	1.61; 5.29	< 0.001*
	No	45	26	1			1		
Salvage surgery	Yes	34	21	0.47	0.28; 0.79	0.004*	0.47	0.25; 0.89	0.020*
	No	66	47	1			1		
Previous RT	Yes	38	30	1.87	1.15; 3.04	0.010*			
	No	63	38	1					
	cont. per Gy	101	68	1.01	1.00; 1.02	0.005*	1.00	0.99; 1.01	0.778
hsmGPS	hsmGPS 0	18	12	1					
	hsmGPS 1	36	26	1.71	0.86; 3.41	0.126			
	hsmGPS 2	4	3	6.69	1.78; 25.11	0.005*			
EQD2_10Gy_	< 50 Gy	23	17	2.16	1.23; 3.77	0.006*			
	≥ 50 Gy	76	49	1					
	cont. per Gy	99	66	0.95	0.93; 0.98	< 0.001*	0.98	0.94; 1.01	0.118

Before applying the univariate and multivariate model, Kaplan–Meier analysis was performed, and the presence of proportional hazards was verified. Included in the multivariate model (*n* =  81, events 53, degrees of freedom 5) were UICC stage, PEG tube, salvage surgery, cumulative dose of previous irradiation at local site during first-line treatment, and current radiation dose EQD2_10Gy_. Significant values are marked with *. PEG: percutaneous endoscopic gastrostomy, RT: radiotherapy, hsmGPS: high-sensitive modified Glasgow prognostic score, EQD2: Dose equivalent to 2 Gy, OS: overall survival, UV: Univariate, MV: multivariate, HR hazard ratio, CI: confidence interval, cont: continuous

We identified patients with PEG tube (HR 2.92, CI 1.61; 5.29, *p* < 0.001) and patients with UICC grade IV tumour (HR 2.11, CI 1.11; 4.02, *p* =  0.023) as independent negative prognostic outcome factors for OS. Additionally, salvage surgery was an independent positive prognostic outcome factor for OS (HR 0.47, CI 0.25; 0.89, *p* = 0.020).

### Adverse events

Both patient groups (initial RT and re-RT) were comparable regarding adverse events. A total of 359 adverse events was documented for all patients with 232 events (3.68 events per patient) in initial RT group and 127 (3.34 events per patient) in re-RT group. Events grade III or higher occurred 44 times (0.69 events per patient) in initial RT group compared to 24 times (0.63 events per patient) for re-RT group. In each group, there was one grade V event of occurring death directly associated to radiotherapy. In the initial RT group, one patient deceased during RCT after experiencing intermittent syncope’s of an unknown cause resulting in cardiac arrest, in re-RT group one patient died of treatment-associated pneumonia during RCT. Grade IV events for initial RT included: Dermatitis *n* =  1, dysphagia *n* =  3 and intensive bleeding *n* =  1; for re-RT: dysphagia *n* =  3. A comprehensive list of CTCAE graded events and other non-severe adverse events that were not documented within the CTCAE structure is shown in Table 4.

**Table 4:** Incidence of adverse events during initial RT/re-RT of patients with locoregional recurrence of head and neck squamous cell carcinoma (LR-HNSCC). Only for selected adverse events, grading according to CTCAE was documented. iRT = initial RT; RT = radiotherapy, CTCAE = Common Terminology Criteria for Adverse Events

Adverse events		iRT (*n* = 63)	re-RT (*n* = 38)
		Total n (% of total per group)	n (% of total per group)
Common adverse events documented within CTCAE			
Mucositis	Any grade	38 (60.3)	20 (52.6)
	CTCAE I-II	29 (46.0)	16 (42.1)
	CTCAE ≥ III	9 (14.3)	4 (10.5)
Dermatitis	Any grade	43 (68.3)	20 (52.6)
	CTCAE I-II	36 (57.1)	19 (50.0)
	CTCAE ≥ III	7 (11.1)	1 (2.6)
Dysphagia	Any grade	42 (66.7)	25 (65.8)
	CTCAE I-II	28 (44.4)	15 (39.5)
	CTCAE ≥ III	14 (22.2)	10 (26.3)
Xerostomia	Any grade	28 (44.4)	15 (39.5)
	CTCAE I-II	25 (39.7)	11 (28.9)
	CTCAE ≥ III	3 (4.8)	4 (10.5)
Lymph oedema	Any grade	22 (34.9)	6 (15.8)
	CTCAE I-II	18 (28.6)	6 (15.8)
	CTCAE ≥ III	4 (6.3)	0 (0.0)
Obstipation	Any grade	6 (9.5)	3 (7.9)
	CTCAE I-II	3 (4.8)	1 (2.6)
	CTCAE ≥ III	3 (4.8)	2 (5.3)
Bleeding	Any grade	5 (7.9)	2 (5.3)
	CTCAE I-II	3 (4.8)	1 (2.6)
	CTCAE ≥ III	2 (3.2)	1 (2.6)
Other severe adverse events CTCAE ≥ III			
Grade V	Cardiac arrest due to syncope’s of unknown origin	1 (1.6)	0
	Pneumonia	0 (0.0)	0
Grade III	Myocardial Infarction	1 (1.6)	0
	Stroke	0 (0.0)	1
	Epileptic seizure	0 (0.0)	1
Other non-severe adverse events not documented within CTCAE			
CTCAE grade unknown	Skin fibrosis Taste impairment	9 (14.3)	9 (23.7)
	Hearing impairment	9 (14.3)	11 (28.9)
	Nerval damage	4 (6.3)	2 (5.3)
	Dental damage	5 (7.9)	2 (5.3)
	Fatigue	4 (6.3)	3 (7.9)
	Conjunctivitis	8 (12.7)	4 (10.5)
	Would healing impairment	1 (1.6)	0 (0.0)
	Acute kidney dysfunction	0 (0.0)	1 (2.6)
	Thrombosis	2 (3.2)	1 (2.6)
	Catheter-associated	1 (1.6)	0 (0.0)
	Infection	1 (1.6)	0 (0.0)
	Tracheitis	1 (1.6)	0 (0.0)
	Herpesvirus infection	1 (1.6)	0 (0.0)

## Discussion

In this study, several prognostic factors were identified as significant predictors of better OS. These included initial RT, high applied radiation dose ≥ 50 Gy, UICC grade I-III, non-inflammatory status as assessed by hsmGPS, feasibility of salvage surgery, and no PEG tube. The factors age, BMI, ECOG-PS, tobacco and alcohol consumption did not significantly affect OS in our cohort.

Initial RT and re-RT continue to play critical roles in the multidisciplinary management of LR-HNSCC. In our analysis, re-RT patients had a significantly poorer OS than initial RT patients (12 months vs. 35 months). A recurrence in a previously irradiated area suggests the existence of a radioresistant clone [35, 36], which in turn could limit the therapeutic effect of re-RT. This problem seems to be particularly relevant in patients previously treated with RCT [37]. In our cohort, subgroup analysis for RCT treated patients showed significantly longer OS for initial RT patients compared to re-RT patients (35 months vs 12 months). Due to the cohort size, detailed subgroup analysis of initial RT vs re-RT analysis for RCT could not be performed but should be assessed in future prospective trials.

To address tumour radio resistance, strategies such as radio sensitizing chemotherapy, dose escalation, alternative fractionation regimens, and brachytherapy are usually employed [36, 38]. Evidence from multiple studies underscores the correlation between higher radiation doses and improved outcomes, particularly in challenging cases of radioresistant tumours [35, 39]. Despite this, our real-world retrospective analysis showed that re-RT patients often receive lower median doses (50 Gy) compared to initial RT patients (63 Gy). Furthermore, we observed that doses below 50 Gy were associated with significantly poorer OS. This discrepancy highlights a critical limitation in the feasibility of achieving high-dose radiation in the re-RT setting. While higher doses are well-documented to improve outcomes, the clinical reality is that these doses are often unattainable due to constraints such as re-RT, patient tolerance, and concerns about severe toxicity. Consequently, the inability to consistently deliver optimal doses emerges as a key barrier to improving outcomes in this patient population.

This study identified recurrent UICC stage IV disease as an independent negative prognostic factor for OS in multivariate analyses. This aligns with findings from other publications, which report that extensive recurrences—characterised by larger tumours and/or multiple nodal involvements—are associated with worse outcomes [40, 41]. These data emphasise that, in line with the literature, the stage and extent of recurrence have a significant impact on patient prognosis, highlighting the critical need for early detection, long and close follow-ups with regular radiological imaging and clinical examination and tailored therapeutic strategies.

Salvage surgery was a major prognostic factor in our study for patients with LR-HNSCC. Surgical debulking of the recurrent tumour has consistently been associated with improved outcomes in the literature [15, 17, 37, 42, 43]. Our findings therefore align with previous evidence that supports a surgical approach whenever feasible. Our findings also revealed that adding adjuvant RT even enhances clinical outcomes. Hence, the use of adjuvant RT in both initial RT and re-RT cases further enhances the therapeutic benefit, suggesting a combined modality approach for optimising outcomes in this challenging population.

In our cohort, patients who had a PEG tube had significantly shorter OS. PEG tube was the strongest negative independent impact factor for OS. Our data suggest that the decision on intensive treatments in patients that either require a PEG due to medical need or that are at high risk for malnutrition and receive a prophylactic PEG should be made carefully. The presumably poorer prognosis should be discussed with the patient and individual goals of therapy should be considered. However, no other (e.g. purely palliative) treatment recommendation can be derived from this. In general, supplemental prophylactic placement of a PEG tube is particularly recommended in cases of significant weight loss, severe pain, dehydration, comorbidities with an increased risk of malnutrition, and pronounced aspiration [44]. Accordingly, an already inserted PEG tube or the likelihood of a PEG tube placement should be included as a possible prognostic factor in future prospective studies. Early screening of patients for malnutrition and the need for PEG placement, and careful consideration of patients who already have a PEG tube inserted for medical reasons, could be an important measure to mitigate severe disease courses and prolong OS with intensified care and supportive measures (e.g. supportive diets). In addition, we recommend that the efficacy of nutritional intervention should be evaluated in further studies.

In our study, patients with an increased systemic inflammatory status (as evidenced by higher hsmGPS scores) had a worse prognosis than patients with a low inflammatory status. The mGPS has been shown to have prognostic value independent of tumour stage in lung, gastrointestinal and renal cancers [45, 46]. One study with more than 20.000 patients showed that an mGPS of 2 was associated with an estimate 160% reduction in both overall and cancer-specific survival independent of tumour site [47]. We used the hsmGPS according to the work of Hanai et. al. which demonstrated a superiority of the hsmGPS compared to the GPS alone for patients with head and neck cancer [19]. The hsmGPS is a powerful and easy-to-asses prognostic factor that should be implemented in the assessment of LR-HNSCC.

Re-RT can increase the risk of complications such as carotid artery stenosis or rupture, osteoradionecrosis, pharyngocutaneous fistulas, non-healing skin ulcers, and spinal cord damage. This risk is likely reduced with modern RT techniques such as IMRT [48]. We therefore assessed differences in adverse events during and after RT for initial and re-RT patients. Our initial RT and re-RT cohorts did not differ significantly regarding use of RT techniques, including IMRT, volumetric arc RT and 3D-conformal RT. In our study, we did not find significantly increased toxicities between the initial RT and re-RT groups. In each group, there was one grade V fatal event occurring during RCT. In our population, treatment-related fatality rate was less than current data suggests for this group where fatalities can approach 10% and incidence of acute and late toxicity ≥ CTCAE grade III can reach up to 50% [49, 50]. In our patient cohort, the most common grade III toxicity was dysphagia with an incidence of *n* =  14 (22%) for initial RT patients versus *n* =  10 (26.3%) for re-RT patients which was statistically non-significant and far below 50%. Grade IV events (mucositis, dermatitis, dysphagia or bleeding) were rare in both groups. We could thus show, that even in a heavily pre-treated group, RT at LR and even re-RT seems feasible if planned carefully. Our approach to prioritize target volumes and discuss potential toxicities with patients on an individual basis is in line with the consensus paper [32] and led to acceptable side effects. However, prospective evidence on re-RT is scarce and our understanding of the underlying radiobiology is poor. More research is needed to better understand the clinical implications and possibilities of re-RT. The ESTRO and EORTC consensus helps to standardise different forms of re-RT [32]. The implementation of this classification in clinical practice and research enhances the understanding of re-RT’s clinical implications and supports cross-study comparisons. Such data can inform future recommendations for re-RT based on clinical evidence. Additionally, nomograms predicting severe toxicity after re-RT — considering patient, disease, and treatment-related factors — are already available, aiding in personalised treatment planning [51].

### Limitations

A single centre analysis has limited generalisability of the results, and they may not be transferable to other facilities. There may be differences in demographics, clinical practice, or available resources that limit applicability. One limitation of this study lies in its retrospective design, which means that not all data points could be assessed. For instance, the ECOG-PS was only available for 63 (62%) of patients. Previous studies have shown that a low ECOG-PS is associated with longer OS in LR-HNSCC patients [18]. In larger cohorts, we would expect to see more substantial differences in outcomes related to ECOG-PS, as patients with a higher ECOG-PS are generally ineligible for more intensive treatments. However, since ECOG-PS was evenly distributed between the initial RT and re-RT groups with no statistically significant difference, we do not anticipate that it had a meaningful impact on the results of this study. This factor should be considered in future prospective trials, which could offer more robust data in comparison with our results.

Due to the cohort size, a detailed subgroup analysis of the impact of prognostic factors on OS by HNSCC tumour entity could not be performed. The most common tumour entities in our cohort were oral cavity and oropharyngeal cancers (initial RT *n* =  44, 69.8%; re-RT *n* =  24, 63.1%), with no significant differences between the initial RT and re-RT groups. However, given the known variations in prognosis across different tumour entities—such as a more favourable prognosis for nasopharyngeal and laryngeal carcinomas [52–54]—future prospective trials should assess these entities in larger subgroups to evaluate the relevance of the prognostic factors identified in this study.

## Conclusions

Salvage surgery at LR, non-UICC stage IV, initial or adequate re-RT dose, absence of a PEG tube, and a low systemic inflammatory status were associated with improved outcomes and acceptable severe toxicities in our analysis. However, future prospective studies are needed to further refine patient selection and validate the role of these markers in optimising RT strategies. Integrating these factors into clinical practice could help guide treatment decisions, ultimately improving outcomes in this prognostically unfavourable patient group with critical need for personalised treatment approaches.

## Supplementary Information

Supplementary Material

Supplementary Material

Supplementary Material

Supplementary Material

Supplementary Material

Supplementary Material

Supplementary Material

## Data Availability

No datasets were generated or analysed during the current study.
