# Supplementary figures and images for: Optimising (re-)irradiation for locally recurrent head and neck cancer: impact of dose-escalation, salvage surgery, PEG tube and biomarkers on oncological outcomes—a single centre analysis
Source: Radiat Oncol. 2025 Jan 2;20:1. doi: 10.1186/s13014-024-02570-y (PMC11697932; doi:10.1186/s13014-024-02570-y)

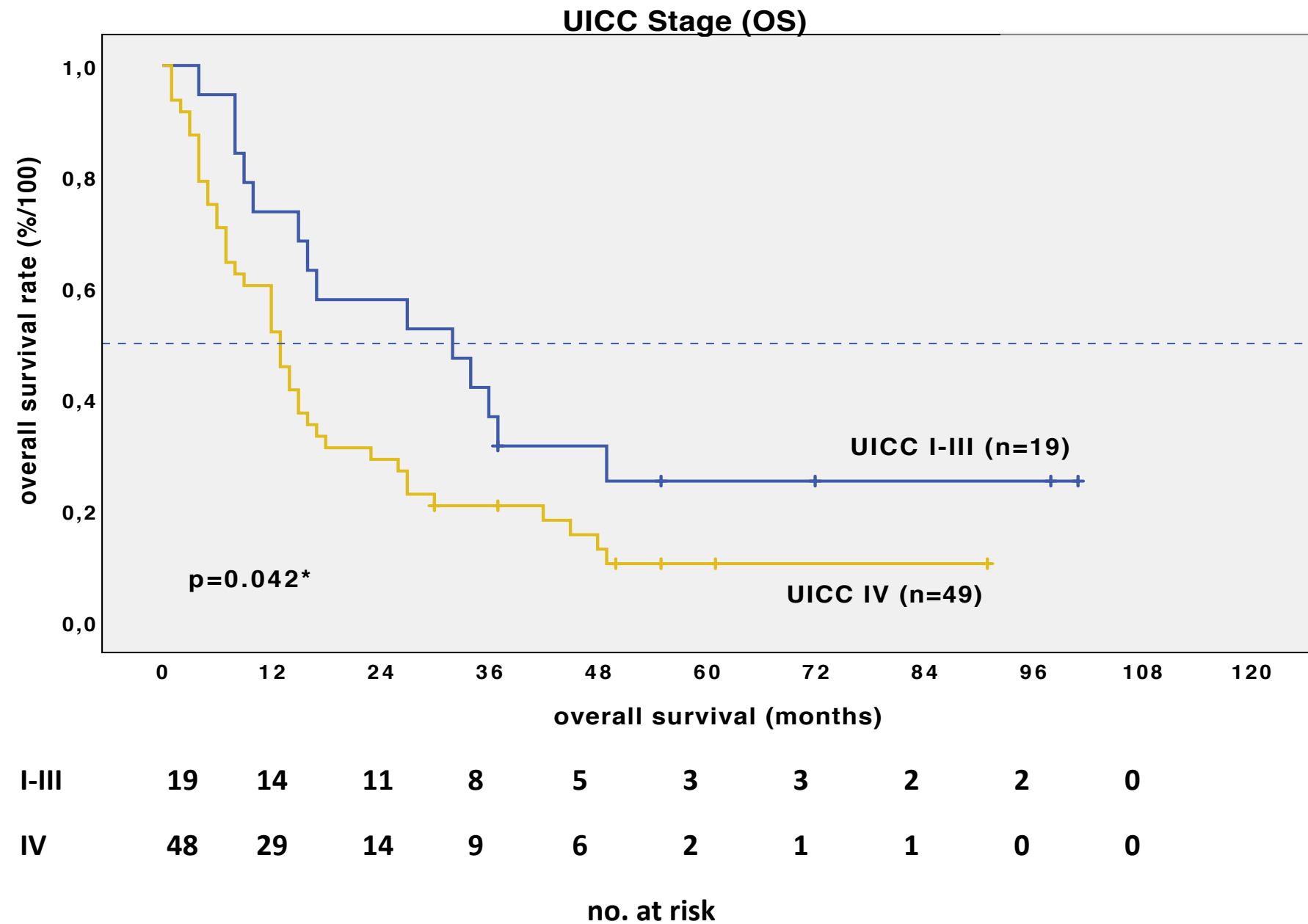

Supplement: Supplementary file 1 — Supplementary Material [file 13014_2024_2570_MOESM1_ESM.pdf]

## PEG tube (OS)

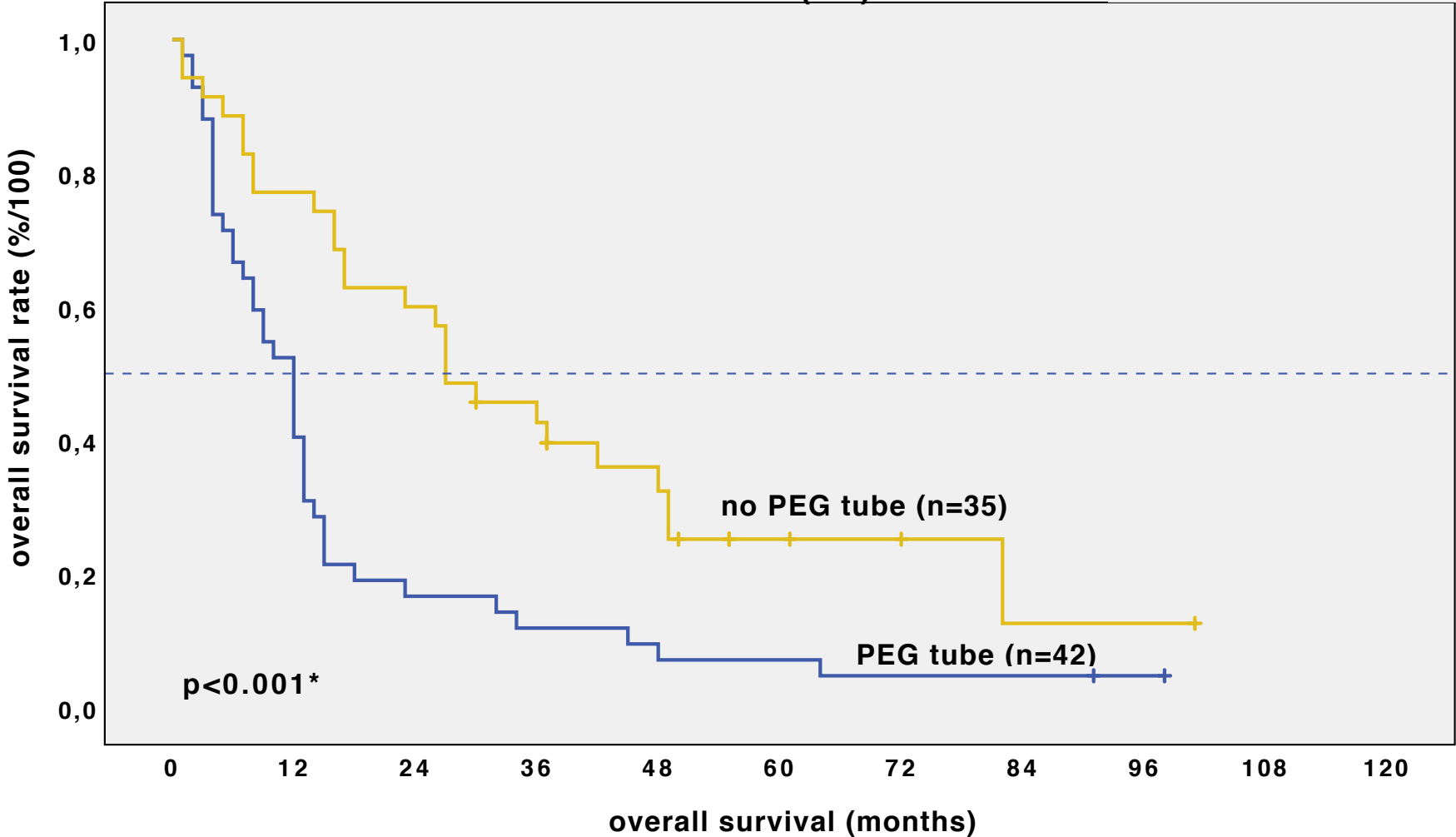

|                  | 0           | 1  | 2  | 3  | 4  | 5 | 6 | 7 | 8 | 9 | 10 |
|------------------|-------------|----|----|----|----|---|---|---|---|---|----|
| Without PEG tube | 35          | 27 | 21 | 15 | 10 | 4 | 3 | 1 | 1 | 0 |    |
| With PEG tube    | 42          | 22 | 7  | 5  | 4  | 3 | 2 | 2 | 1 | 0 |    |
|                  | no. at risk |    |    |    |    |   |   |   |   |   |    |

Supplement: Supplementary file 2 — Supplementary Material [file 13014_2024_2570_MOESM2_ESM.pdf]

\_\_\_\_\_

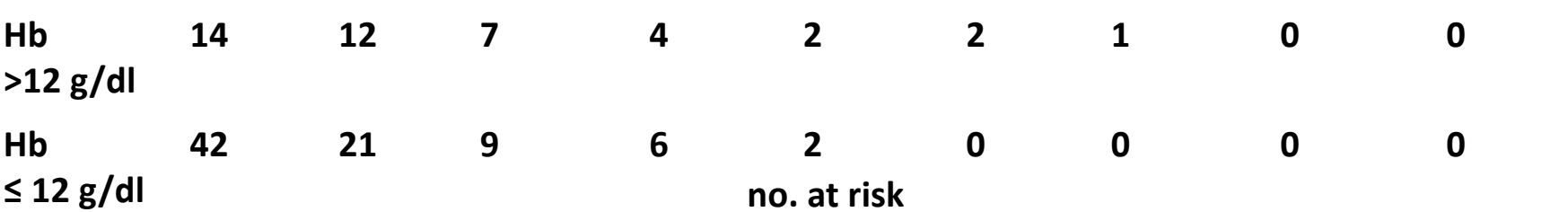

Supplement: Supplementary file 7 — Supplementary Material [file 13014_2024_2570_MOESM7_ESM.pdf]
