# Supplementary material for: Optimising (re-)irradiation for locally recurrent head and neck cancer: impact of dose-escalation, salvage surgery, PEG tube and biomarkers on oncological outcomes—a single centre analysis
Source: Radiat Oncol. 2025 Jan 2;20:1. doi: 10.1186/s13014-024-02570-y (PMC11697932; doi:10.1186/s13014-024-02570-y)
Supplement: Supplementary file 3 — Supplementary Material [file 13014_2024_2570_MOESM3_ESM.pdf]

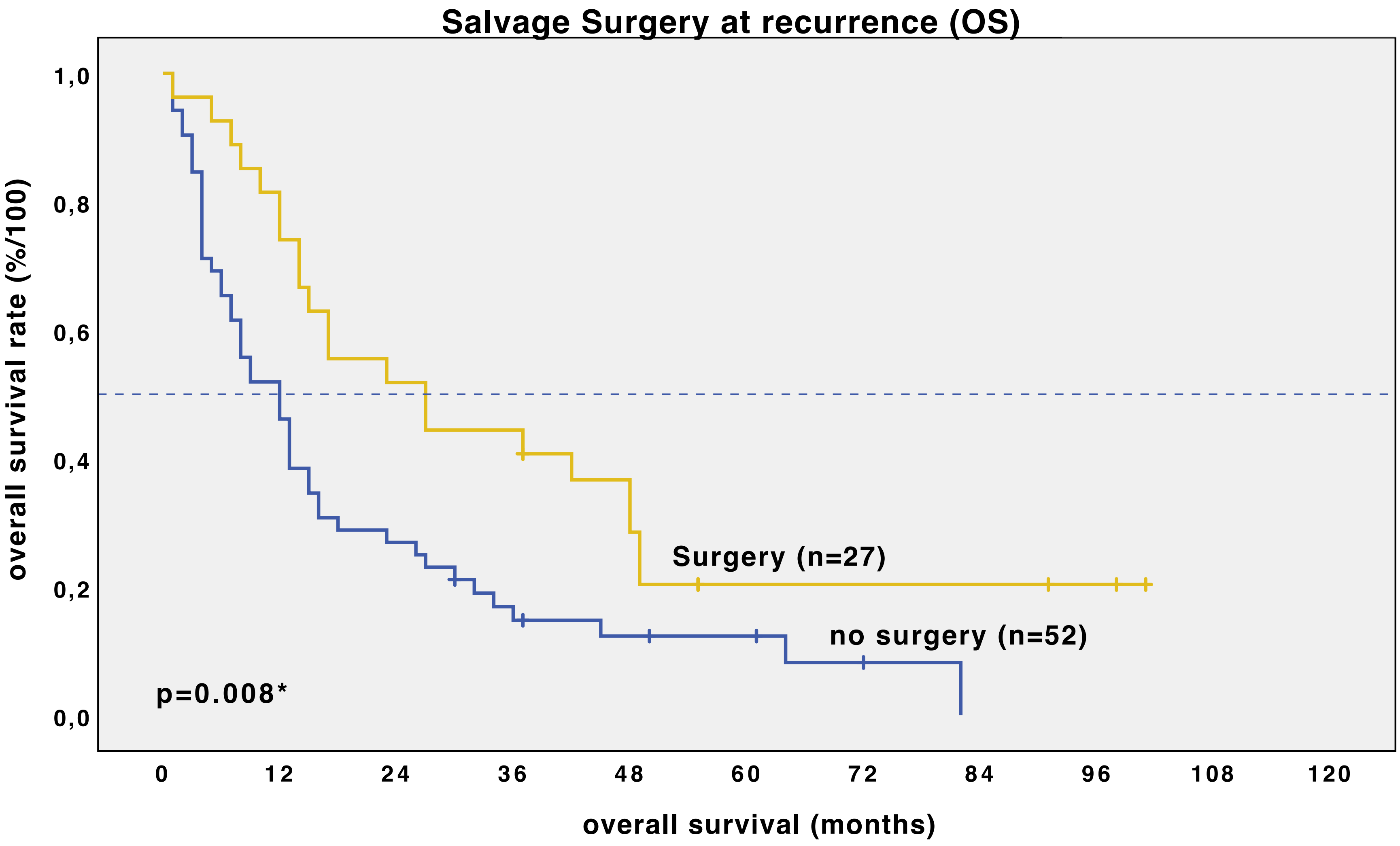

With surgery	27	22	14	12	9	3	3	3	2	0
Without surgery	52	27	14	8	5	4	2	0	0	0

no. at risk
