# Supplementary material for: Optimising (re-)irradiation for locally recurrent head and neck cancer: impact of dose-escalation, salvage surgery, PEG tube and biomarkers on oncological outcomes—a single centre analysis
Source: Radiat Oncol. 2025 Jan 2;20:1. doi: 10.1186/s13014-024-02570-y (PMC11697932; doi:10.1186/s13014-024-02570-y)
Supplement: Supplementary file 6 — Supplementary Material [file 13014_2024_2570_MOESM6_ESM.docx]

**Supplementary Table 1. Baseline Characteristics for LR-HSNCC**

|  |  | LR-HNSCC (n=101) | | |
| --- | --- | --- | --- | --- |
|  |  | n (%) / med (IQR) | | |
| Patient characteristics | | |  |  |
| Sex | Male  Female | 74 (73)  27 (27) | | |
| Age |  | 63 | | |
| ECOG-PS | 0  1  2  3 | 40 (61.5)  16 (24.6)  6 (9.2)  3 (4.6) | | |
| BMI | < 18.5  18.5-24.9  >25.0 | 10 (12.2)  39 (47.6)  33 (40.2) | | |
| Smoking | Yes  No | 58 (69.0)  16 (31.0) | | |
| Alcohol consumption | Substantial (C2 abuse)  Occasionally  None | 15 (18.5)  26 (32.1)  40 (49.4) | | |
| Tumour characteristics | | |  |  |
| Tumour entity | Oral cavity  Nasopharyngeal  Oropharyngeal  Hypopharyngeal  Salivary glands  Laryngeal  Nasal cavity and paranasal sinus  CUP  multilevel | 45 (44.6)  1 (1.0)  23 (0.23)  7 (6.9)  1 (1.0)  17 (16.83)  2 (2.0)  1 (1.0)  4 (4.0) | | |
| UICC stage | I  II  III  IV | 9 (10.6)  8 (9.4)  10 (11.8)  58 (68.2) | | |
| Risk group | Locally limited  Locally advanced | 17 (20.0)  68 (80.0) | | |
| Radiation characteristics | | |  |  |
| Therapy group | RT  RCT  RIT | 37 (36.6)  45 (44.6)  19 (18.8) | | |
| Technique | IMRT  VMAT  3D-CRT | 27 (27.0)  28 (28.0)  45 (45.0) | | |
| EQD2_10Gy_ | Gy | 60 | | |
| Dose per fraction | Gy | 1.8 (0.2) | | |
| Inclusion of lymph drainage region | Yes  No | 58 (57.4)  43 (42.6) | | |
| Additional therapies | | |  |  |
| Chemotherapy | Cisplatin/Carboplatin mono  Cisplatin/5-FU  Carboplatin/Paclitaxel  Others | 19 (46.3)  3 (7.3)  15 (36.6)  4 (9.6) | | |
| Salvage surgery | Yes  No | 66 (66.0)  34 (34.0) | | |
| Curative intent | Yes  No | 70 (70.0)  30 (30.0) | | |

**Supplementary Table 1: Baseline Characteristics for LR-HSNCC**. Reported n are valid n. Med = median; IQR = interquartile range; ECOG-PS = Eastern Cooperative Oncology Group Performance Score; BMI = body mass index; RT = radiotherapy; RCT = radio-chemotherapy; RIT = radioimmunotherapy; IMRT = intensity-modulated RT; VMAT = Volumetric Intensity Modulated Arc Therapy; 3D-CRT = three-dimensional conformational RT; EQD2 = equivalent dose of 2Gy, PTV = planned target volume, Gy = Gray.
