# Supplementary material for: Optimising (re-)irradiation for locally recurrent head and neck cancer: impact of dose-escalation, salvage surgery, PEG tube and biomarkers on oncological outcomes—a single centre analysis
Source: Radiat Oncol. 2025 Jan 2;20:1. doi: 10.1186/s13014-024-02570-y (PMC11697932; doi:10.1186/s13014-024-02570-y)
Supplement: Supplementary file 4 — Supplementary Material [file 13014_2024_2570_MOESM4_ESM.pdf]

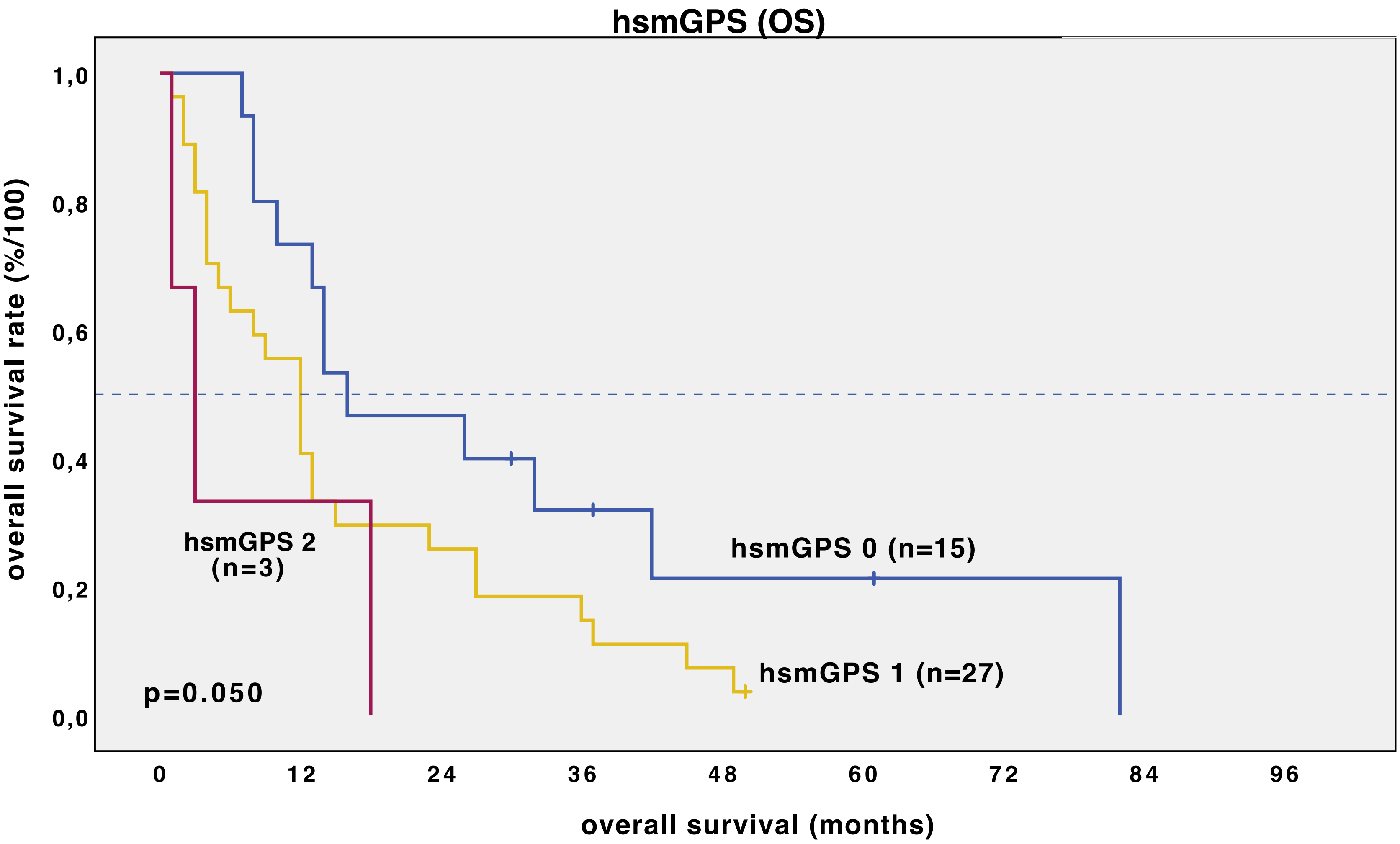


| | | | | | | | | | |
|---|---|---|---|---|---|---|---|---|---|
| hsmGPS 0 | 15 | 11 | 7 | 4 | 2 | 2 | 1 | 0 | 0 |
| hsmGPS 1 | 27 | 15 | 7 | 5 | 2 | 0 | 0 | 0 | 0 |
| hsmGPS 2 | 3 | 1 | 0 | 0 | 0 | 0 | 0 | 0 | 0 |

no. at risk
