# Supplementary material for: Optimising (re-)irradiation for locally recurrent head and neck cancer: impact of dose-escalation, salvage surgery, PEG tube and biomarkers on oncological outcomes—a single centre analysis
Source: Radiat Oncol. 2025 Jan 2;20:1. doi: 10.1186/s13014-024-02570-y (PMC11697932; doi:10.1186/s13014-024-02570-y)
Supplement: Supplementary file 5 — Supplementary Material [file 13014_2024_2570_MOESM5_ESM.pdf]

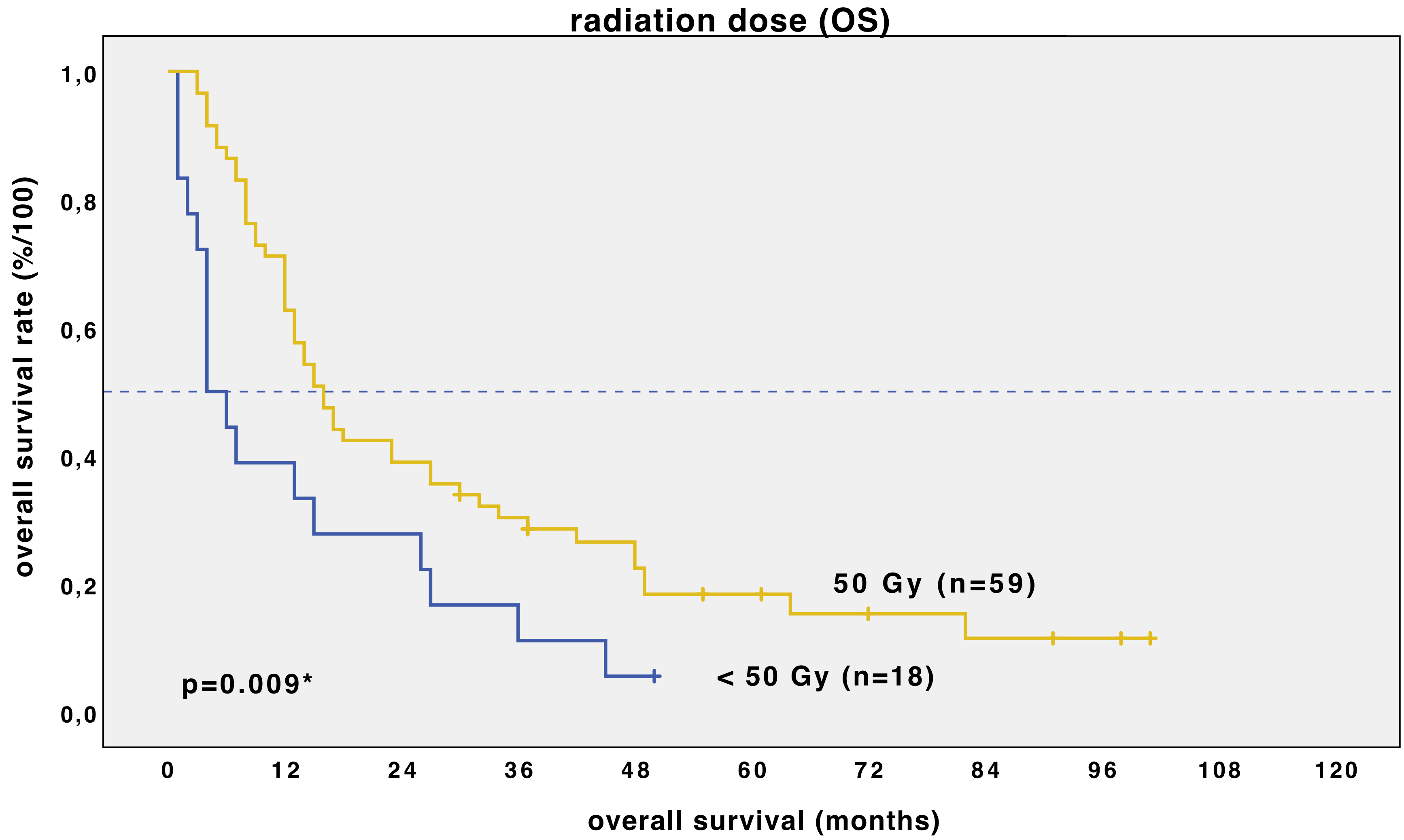

≥50Gy	59	42	23	17	13	7	5	3	2	0
<50Gy	18	7	5	3	1	0	0	0	0	0

no. at risk
